# A pooling-based genome-wide analysis identifies new potential candidate genes for atopy in the European Community Respiratory Health Survey (ECRHS)

**DOI:** 10.1186/1471-2350-10-128

**Published:** 2009-12-06

**Authors:** Francesc Castro-Giner, Mariona Bustamante, Juan Ramon González, Manolis Kogevinas, Deborah Jarvis, Joachim Heinrich, Josep-Maria Antó, Matthias Wjst, Xavier Estivill, Rafael de Cid

**Affiliations:** 1 Centre for Research in Environmental Epidemiology (CREAL), Barcelona, Spain; 2 Municipal Institute of Medical Research (IMIM-Hospital del Mar), Barcelona, Spain; 3 Public Health and Epidemiology Network Biomedical Research Center (CIBERESP), Barcelona, Spain; 4 Genes and Disease Program, Center for Genomic Regulation (CRG), Barcelona, Spain; 5 Medical School, University of Crete, Heraklion, Greece; 6 Respiratory Epidemiology and Public Health Group, National Heart and Lung Institute, Imperial College, London, UK; 7 Institute of Epidemiology, Helmholtz Zentrum München, Munich, Germany; 8 Department of Health and Experimental Sciences, University Pompeu Fabra, Barcelona, Spain; 9 German Research Center for Environmental Health, Helmholtz Centre GSF, Munich, Germany; 10 CEA, Institute de Genomique. Centre National de Genotypage (CNG), Evry, France

## Abstract

**Background:**

Asthma and atopy are complex phenotypes with shared genetic component. In this study we attempt to identify genes related to these traits performing a two-stage DNA pooling genome-wide analysis in order to reduce costs. First, we assessed all markers in a subset of subjects using DNA pooling, and in a second stage we evaluated the most promising markers at an individual level.

**Methods:**

For the genome-wide analysis, we constructed DNA pools from 75 subjects with atopy and asthma, 75 subjects with atopy and without asthma and 75 control subjects without atopy or asthma. In a second stage, the most promising regions surrounding significant markers after correction for false discovery rate were replicated with individual genotyping of samples included in the pools and an additional set of 429 atopic subjects and 222 controls from the same study centres.

**Results:**

*Homo sapiens *protein kinase-like protein SgK493 (*SGK493*) was found to be associated with atopy. To lesser extent mitogen-activated protein kinase 5 (*MAP3K5*), collagen type XVIII alpha 1 (*COL18A1*) and collagen type XXIX alpha 1 (*COL29A1*) were also found to be associated with atopy. Functional evidences points out a role for *MAP3K5*, *COL18A1 *and *COL29A1 *but the function of *SGK493 *is unknown.

**Conclusion:**

In this analysis we have identified new candidate regions related to atopy and suggest *SGK493 *as an atopy locus, although these results need further replication.

## Background

Asthma and atopy are complex phenotypes with environmental and genetic determinants. Several chromosomal regions and candidate genes have been implicated in asthma or atopy susceptibility [1,2]. Genome wide association (GWA) studies have been successful in the identification of loci contributing to complex diseases, included asthma [3-5]. However a limitation of GWA studies is the high economic cost required. A cost-effective alternative is the use of pooled DNA followed by individual genotyping [6-8]. In this study, we performed a pooling-based GWA for asthma and atopy, with further validation of most promising regions in the individual pooled samples as well as in a second set of cases and controls.

## Methods

### Pooling GWA

In the first stage we conducted a GWA analysis using a DNA-pooling approach. Genotyping was performed using the Illumina HumanHap 300 Whole-Genome Genotyping BeadChip. The genome-wide analysis was conducted in three different sets of pooled samples: (1) subjects with atopy and asthma; (2) subjects with atopy and without asthma, and (3) control subjects with neither asthma nor atopy (Table 1). Samples for this analysis were randomly selected from United Kingdom, Spain and Germany cohorts of the European Community Respiratory Health Survey (ECRHS) study [9]. Atopy was defined as sensitization (IgE levels >0.35 kU/L) to specific allergens (*D. Pteronyssinus*, cat, timothy grass. or *C. herbarum*). Asthma was defined as the presence of attacks of asthma in the last 12 months or taking currently medication for asthma. All asthmatics subjects had also atopy. For pool construction, DNA samples were diluted and measured twice by double stranded DNA quantification using PicoGreen^® ^dsDNA reagent Kit (Invitrogen), and then normalized at 50 ng/μl. After visual inspection in 0.7% alkaline agarose gels, selected DNA samples were pooled in three independent pools of 75 individuals each one. We have controlled the quality of the Illumina genotyping process including a sample from HapMap (GM12873) with known genotype for the panel of 300K markers. We verified the concordance of genotypes with the HapMap database (concordance = 99.91%) and inconsistent single nucleotide polymorphisms (SNPs) were excluded from the analysis (n = 296, 0.09%). A pool of HapMap (n = 57) subjects were also performed and genotyped to validate the allele frequency estimation. SNPs with a poor performance in the estimation of allele frequency were excluded from the analysis (n = 3271, 1.03%) using a threshold of > 12% of difference, as shown in previous reports [7,10], Finally, three replicates of each pool of ECRHS subjects were constructed to account for sampling errors in the analysis.

**Table 1:** Population characteristics of asthma and atopy analyzed samples

	Pooling Samples			Replication samples		
	**Control**	**Atopy**	**Atopic asthma**	**Control**	**Atopy**	**Atopic asthma subsample**

Subjects, n	75	75	75	222	429	198

Subjects excluded*, n (%)	14 (19)	17 (23)	14 (19)	47 (21)	98 (23)	48 (24)

Age, mean (sd)	35.87 (6.79)	32.21 (7.78)	32.07 (6.95)	35.36 (7.16)	33.32 (7.15)	33.19 (7.14)

Gender						

Males, n (%)	37 (49)	38 (51)	37 (49)	106 (48)	205 (48)	84 (42)

Females, n (%)	38 (51)	37 (49)	38 (51)	116 (52)	224 (52)	114 (58)

Smoking						

Current, n (%)	19 (25)	23 (31)	28 (38)	56 (25)	100 (23)	37 (19)

Ex, n (%)	20 (27)	18 (24)	16 (22)	58 (26)	96 (22)	47 (24)

Never, n (%)	36 (48)	34 (45)	30 (41)	107 (48)	232 (54)	114 (58)

*Subjects excluded at the individual genotyping (stage 2)

Allele frequencies for each SNP were estimated from pooled samples correcting by the ratio of intensity of both alleles [11]. Allele frequencies in each pool were compared using the 1 df chi-square T statistic for testing differences between two proportions accounting for experimental and sampling errors [11]. Multiple testing was controlled using a false discovery rate (FDR) at 5% [12]. Allele frequency estimation was validated using a pool of 57 samples from HapMap whose individual genotypes for 300K Illumina panel were previously known. Predicted and real allele frequencies showed a strong correlation (Pearson correlation coefficient, r = 0.99).

### Individual genotyping in ECHRS

In a second stage, replication was performed by individual genotyping of subjects included in the pools (n = 225) and in an additional set of 429 atopic subjects (46% with asthma) and 222 controls subjects randomly selected from the ECRHS study. Only promising SNPs that were still significant after correction by multiple testing were followed up. Target regions were delimited by non significant SNPs (p > 0.05) upstream and downstream from the SNP significant associated after multiple testing corrections in the first stage (see Additional file 1: table S1). Complex regions with genomic structural variants were discarded for this study (chr 1 and chr 5) [13]. For the saturation we used a tag SNP approach forcing the inclusion of the polymorphisms detected in the first stage, or their perfect tags (r^2 ^≥ 0.8). From the HapMap project data set, we utilized genotypes from the public release 21a (phase II; NCBI35, dbSNPb125) corresponding to 90 individuals from the CEPH 30 trios of European descent. The selection of tag SNPs was performed using the pair-tagging strategy implemented in Haploview software (v.4.0) with a MAF ≥ 0.05 and an r^2 ^≥ 0.8 [14]. In addition to HapMap SNPs, four other polymorphisms previously described to be associated with atopic dermatitis and situated in one of the targeted regions (*COL29A1*) were included [15]. The 53 SNPs selected were genotyped in individual samples using SNPlex technology (Applied Biosystems).

Genotyping quality was controlled by including negative controls and internal positive controls consisting in four replicates of two HapMap reference samples. Both, genotype concordances with the HapMap database and among replicates, were verified. Genotype data caring was done using *SNP analysis to results *software (SNPator) http://www.snpator.com/. Forty-five out of 225 pooled samples and 145 out of 651 replication samples were dropped from the individual analysis due to low volume of DNA available or low concentration (Table 1). Seven out of 53 genotyped SNPs were excluded from the analysis since deviation from Hardy-Weinberg equilibrium (HWE) (in controls p < 0.05) or showed a genotyping rate under 80%. The final replication set included 46 SNPs with an overall genotyping rate of 97% in 686 samples (Table 1). Genotyping assays were performed at the Barcelona Node of the "Centro Nacional de Genotipado" (CeGen) in Spain.

The statistical analysis for data derived from individual genotyping was performed assuming an additive genetic model and using logistic regression using R statistical software package [16]. Models were adjusted for age, sex and smoking status. Country of origin was considered in the regression models in order to discard confounding by population stratification [17]. Linkage disequilibrium (LD) blocks were estimated according to Gabriel et al. [18] method implemented in Haploview.

### SGK493 tissue expression

A human RNA tissue panel (Stratagene) including brain, heart, kidney, liver, lung, placenta, skeletal muscle, spleen, testis, ovary and thymus was used to evaluate the expression of *SGK493*. One μg of RNA was converted to cDNA using the SuperScript III First-Strand Synthesis System for RT-PCR (Invitrogen). *SGK493 *and the GAPDH genes were amplified in independent PCRs using the following primers *SGK493*-F: 5'-AAGTGACGGACCTGGATGAC-3' and SGK493-R: 5'-TGTGAGGCAGGAGGTATGTG-3' and GAPDH-F: 5'-ACCCAGAAGACTGTGGATGG-3' and GAPDH-R: 5'-TGCTGTAGCCAAATTCGTTG-3'. The expected fragment sizes according to hg18 are 189 bp and 415 bp, respectively. The PCR was performed on a final volume of 25 μl using 0.5 U of BioTherm DNA polymerase (GeneCraft), 2 mM of Mg, 50 μM of each dNTP, 0.12 μM of each primer and 1 μl or 0.5 ul of cDNA (*SGK493 *and GAPDH, respectively). Finally 10 μl of the *SGK493 *PCR and 5 μl of the GAPDH PCR were resolved on a 2% agarose gel.

The study was conducted in accordance with the Declaration of Helsinki Principles. Ethical approval was obtained for each centre from the appropriate institutional ethics committee and all subjects participating in this study provided informed consent.

## Results

### Pooling GWA

A genome wide analysis was performed with three pools of DNA from atopic, atopic asthmatics and control individuals. Population characteristics are shown in Table 1. Significant results after correction for 5% FDR in the GWA of pooled DNA are shown in Table 2 (see Additional file 1: table S1 for the genome-wide surrounding SNPs). Seven SNPs were associated with atopy and one with atopic asthma. From these results, five regions were selected for individual genotyping at a second stage. Three of these regions contained genes putatively related to asthma or atopy: collagen type XXIX alpha 1 (*COL29A1*, also known as *COL6A5*, a new member of the collagen family [19]), mitogen-activated protein kinase kinase kinase 5 (*MAP3K5*, also known as apoptosis signal-regulating kinase 1 (*ASK1*)), collagen type XVIII alpha 1 (*COL18A1*) and solute carrier family 19 member 1 (*SLC19A1*). The other two regions contained genes for which no obvious functionality could be inferred: *Homo sapiens *protein kinase-like protein SgK493 (*SGK493*) and a predicted gene in chromosome 8 (NT_007995.50) which presented two consecutive SNPs associated with atopy in the pooled analysis. Additional SNPs surrounding these six markers were genotyped at an individual level (see Additional file 1: table S2, for the results of the 53 SNPs genotyped at an individual level).

**Table 2:** SNPs associated with asthma or atopy with a FDR of 5% in the pooling-based GWA

SNP	Chromosome	Position	Comparison	p value	fAco-fAca	Region	Saturation
rs2501618	1	178225432	Atopy vs. controls	1.26 × 10^-7^	0.26	*CEP350*	No

rs4952590	2	42130425	Atopy vs. controls	4.63 × 10^-7^	0.19	*SGK493 (LOC91461)*	Yes

rs7629719	3	131642624	Atopy-Asthma vs. controls	1.11 × 10^-7^	0.21	*COL29A1 (COL6A5)*	Yes

rs9292961	5	20975886	Atopy vs. controls	5.38 × 10^-7^	0.3	Segmental Duplications	No

rs9376221	6	137034072	Atopy vs. controls	3.94 × 10^-7^	0.25	*MAP3K5 (ASK1)*	Yes

rs7843085	8	33235086	Atopy vs. controls	3.93 × 10^-7^	0.26	NT_007995.50	Yes

rs13273924	8	33246541	Atopy vs. controls	7.56 × 10^-7^	0.26	NT_007995.50	Yes

rs2330183	21	45777720	Atopy vs. controls	1.59 × 10^-7^	0.28	*SLC19A1*	Yes

fAco (allele frequency in controls); fAca (allele frequency in cases); *CEP350*: centrosomal protein 350 kDa; *SGK493*: protein kinase-like protein SgK493; *COL29A1*: collagen, type XXIX, alpha 1; *MAP3K5*: mitogen-activated protein kinase kinase kinase 5; *ASK1*: apoptosis signal-regulating kinase 1; *SLC19A1*: solute carrier family 19 (folate transporter), member 1

### Individual genotyping

A summary of the positive results on individual samples for atopy is shown in Table 3. SNPs found to be significant for atopy at individual level in both pooling samples and replication set were located in *SGK493 *and *MAP3K5 *genes. Variants in *COL18A1 *and *COL29A1 *were not consistently replicated in both samples, although a variant in the *COL18A1 *(at 5' of the *SLC19A1 *region) was significant in pooling samples (p = 0.005) and border significant in the replication (p = 0.06). For variants listed in Table 3, the combined analysis of samples from pooling and the additional case control set, led to lower p values, indicating that genetic effects are in the same direction in both sets [20].

**Table 3:** Summary of the SNPs significantly associated with atopy at an individual level

					ECHRS Atopy vs. controls		
**SNP**	**Gene**	**MAF**	**Chromosome**	**Position (build36)**	**Pooling**	**Replication**	**Combined**

rs11124858*	*SGK493*	0.367	2	42113828	0.0021	0.0318	0.0007^†^

rs13409978*	*SGK493*	0.122	2	42119206	0.0007^†^	0.0124	0.0001^†^

rs4952590**	*SGK493*	0.141	2	42130425	0.0001^†^	0.0008^†^	1.9 × 10^-6†^

rs1440095	*SGK493*	0.379	2	42143170	0.0098	0.1078	0.0105

rs10934938	*COL29A1*	0.23	3	131596325	0.1158	0.0291	0.0056

rs9402839*	*MAP3K5*	0.138	6	137036903	0.0355	0.0524	0.0099

rs9494554**	*MAP3K5*	0.095	6	137038675	0.0306	0.0035	0.0005^†^

rs12483377*	*COL18A1*	0.108	21	45755537	0.0046	0.0607	0.0030

*SNPs found associated in the pooling-based GWA or their perfect tags (r^2 ^= 0.8) used as substitutes in the genotyping design.

**SNPs that remained significant after a 5% FDR in the pooling-based GWA

^†^**P-values that remain significant after Bonferroni correction (46 SNPs, p ≤ 0.001)**

**Figure 1:**
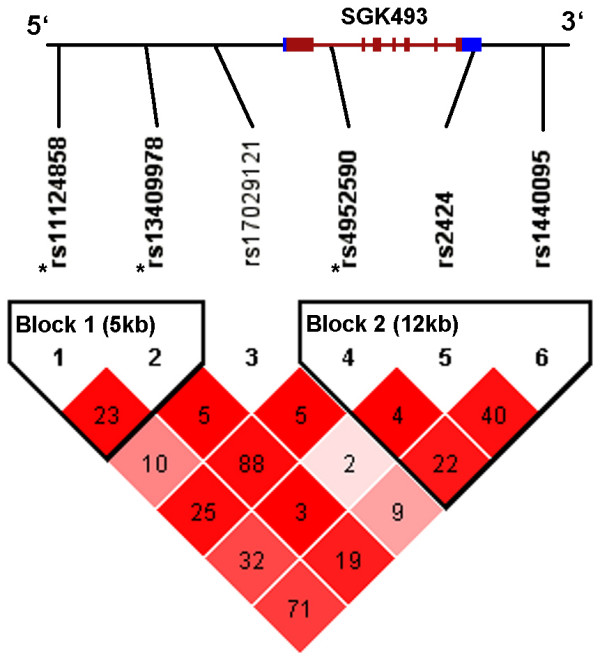
**Linkage disequilibrium pattern and haplotype blocks of SNPs in the *SGK493 *gene using genotyping data in this study**. Colors correspond to D' and numbers in each cell to r^2 ^parameter. Blocks were defined with method described by Gabriel et al. [18]. In red is marked the coding region of the *SGK493 *gene (lines are introns and boxes exons) and blue boxes show the untranslated (UTR) regions of the gene. * SNPs found associated in the individual pooled samples and replication sample (Table 3).

**Figure 2:**
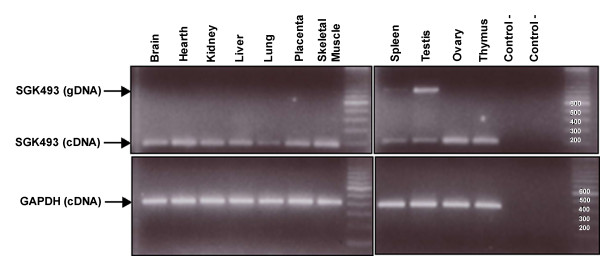
***SGK493 *expression in a panel of human tissues**. Primers are situated in exon 3 and 4 according to the RefSeq Genes. All the human tissues tested express *SGK493*. The amplification of *GAPDH *has been used as a control of the cDNA quality. In spleen and testis a second fragment of approximately 800 bp is observed. This fragment, based on its size, could be the amplification of *SGK493 *from genomic DNA.

The region in chromosome 2 containing the *SGK493 *gene showed the SNPs with the most significant p values in the association with atopy, and similar results were observed for the subset of atopic individuals with asthma (see Additional file 1: table S2). The variant rs4952590 remain significant even after Bonferroni correction (46 SNPs, p ≤ 0.001) in the both pooling and replication samples. This variant was associated with a reduction in risk of atopy in both samples (odds ratio (OR) = 0.18, 95% confidence interval (CI) 0.07-0.47 for pooling sample and OR = 0.52, 95%CI 0.35-0.79 for replication sample). In addition, variant rs13409978 in the pooling sample and three SNPs (rs11124858, rs13409978, rs4952590) remained significant in the combined analysis after Bonferroni correction. Figure 1 shows the representation of the linkage disequilibrium (LD) pattern for the 6 SNPs of *SGK493 *genotyped for all individuals of the sample (n = 699). Two blocks of disequilibrium were defined according to Gabriel et al. [18] method implemented in Haploview. Complete LD pattern of the region derived from HapMap data is summarized in the Additional file 1: figure S1. Haplotype analysis using the six SNPs (Table 4) confirms the results obtained in the single marker analysis. The only haplotype containing C allele of rs13409978 and T allele of rs4952590 is the only significantly associated with atopy (p = 0.00005). These SNPs are in high LD (D'= 0.97 and r^2 ^= 0.88). The expression pattern of *SGK493 *presented in Figure 2 shows that *SGK493 *is ubiquitously expressed in all the human tissues analyzed.

**Table 4:** Haplotype analysis and multiple regression for SGK493 gene variants.

rs11124858	rs13409978	rs17029121	rs4952590	rs2424	rs1440095	Frequency	p-value
A	A	A	C	A	T	0.36	reference

*	*	G	*	*	*	0.22	0.128

G	*	*	*	T	C	0.17	0.506

G	C	*	T	*	C	0.12	0.00005

* Same allele to reference haplotype (most frequent)

The intronic SNP rs9494554, located within *MAP3K5*, was associated with atopy in the pooling and replication samples, and remained significant in the combined analysis after Bonferroni correction. A similar association for rs9494554 was observed in the subsample of atopic asthmatic individuals (see Additional file 1: table S2). Other SNP in *MAP3K5 *(rs9402839) was nominally significant for atopy in pooling sample and border significant in the replication step.

## Discussion

We performed a two-stage DNA pooling-based GWA for asthma and atopy, evaluating a genome-wide panel of markers in a subset of subjects using a DNA pooling strategy, and in a second stage we evaluated the most promising markers at an individual level in the individual pooled samples as well as in a second set of cases and controls. We identified *SGK493 *as a new potential gene associated to atopy. *MAP3K5, COL18A1 *and *COL29A1 *genes were found to lesser extent associated with atopy.

The most significant results were observed for *SGK493 *gene, in chromosome 2, with similar results for atopy and for atopic asthma, probably due to sharing predisposing factors [1]. Significant polymorphisms were located in two different linkage disequilibrium blocks. The first block includes the putative promoter region of *SGK493 *(5' upstream) and the second block covers part of the gene and the 3' untranslated region (UTR) and 3'downstream region (Figure 1). Haplotype based analysis confirmed results obtained in single marker analysis. We could not elucidate which is the functionality of these SNPs and a fine mapping of the region within a more powered sample would be required. We cannot exclude that the functional variant could be tagged by these SNPs and located in another close gene. *SGK493 *gene was identified during the creation of a catalogue of human protein kinases [21], but its particular function is unknown. *SGK493 *could be involved in pathological states as protein kinases mediate most of the signal transduction in eukaryotic cells [22]. We reported that *SGK493 *gene is ubiquitously expressed in human tissues, however a higher expression on lung and uterus was previously identified by microarray experiment from 73 human tissues http://biogps.gnf.org/[23]. Recently, the knock out of *Sgk493 *(also known as *Pkdcc*) in mouse has been described presenting extreme phenotypes that are not *a priori *related with atopy or asthma. The knock out mice showed abnormal respiration and died within a day possibly due to cleft palate [24].

Another kinase *MAP3K5 *was also related to atopy as well as atopic asthma in this study. *MAP3K5 *encodes for a member of the mitogen-activated protein kinase family that regulates the activation of the transcription factor activator protein-1 (AP1) in leukotriene D (4) (LTD(4)) stimulated airway smooth muscle cells and in nitric oxygen (NO) stimulated bronchial epithelial cells [25,26]. AP1 play a role in the production of airway inflammation [26].

Polymorphisms in *COL18A1 *and *COL29A1 *were not consistently replicated. The *COL18A1 *polymorphism shown to be significantly associated with atopy in the pooling sample is located at the 3' coding region of the gene. *COL18A1 *encodes a protein expressed in epithelial and endothelial basement membranes, involved in regulation of angiogenesis and endothelial cell proliferation [27-29]. The *COL29A1 *polymorphism was previously found to be associated with atopic dermatitis [15]. Finally, a region situated in chromosome 8, which contains a predicted gene (NT_007995.50), was also analyzed in detail. Nominally associations were observed in the pooling sample for atopy, but they were not replicated.

Signals described in previous GWA for atopy, asthma and related phenotypes have not been detected in this study [3-5]. The lack of replication of the GWA results can be caused by differences in the definition used to classify affected individuals, genetic coverage of the genome and the p value threshold [30]. In particular, the region detected by Moffatt et al[3] on chromosome 17 has been associated with childhood onset asthma, while in this study the asthmatic individuals were selected independently of their asthma onset. We have not detected the *FCER1A *region identified by Weidinger et al[5] for atopy, but a different genotyping platform was used.

Despite significant replication values of most promising SNPs found in the GWA in pooled DNA, we acknowledge some limitations as the lack of replication for some of the loci identified in the pooling based analysis. Non-replication of the initial findings is a common feature of the initial findings in GWA studies [31], mainly due to heterogeneity in the aetiology of the disease and biases. In addition, the impossibility of detection and adjustment by potential confounders in the analysis of DNA pooling could produce the inconsistencies observed.

Another of the main limitation is the sample size for pool construction and replication. Regarding DNA pooling, the method for measure allele frequency differences corrects by the number of subjects included in each pool [11]. After this correction we obtained some signals at very low p-values, which reinforce the strength of these results. Given limited sample, our study was able to detect variants with larger effects in this population and probably other variants with smaller effects would not be detected. False positives are controlled in the replication phase by individual analysis. Other pooling strategies such as use of different sub-samples of pools would allow capture more biological variation. We acknowledge that with having larger pools we may be losing positive signals but we consider this as a less crucial issue for this analysis. Power calculation shows that replication sample was powered to detect reported associations given the parameters observed in the analysis of individual pooling data (see Additional file 1: table S3). For this reason, some of the results were replicated and significance for a SNP in *SGK493 *reaches Bonferroni level. Although this type of correction by multiple testing is over-conservative and induces false negatives, it is a good indicator of the significance level. In addition to the significance level, some of the results were replicated in the additional sample and replication is considered essential to establish the validity of associations [32].

Finally, another limitation is the small number of regions analyzed in detail in the individual analysis. We discarded complex regions with segmental duplications and putative insertions or deletions [13] because their complexity could *a priori *result in reducing power to detect associations due to non-classical inheritance patterns of markers located on them. In contrast, disease definition is a positive feature of this study since it is highly homogeneous among patients derived from different ECHRS centre, and this is a keystone for valid inferences in association studies.

## Conclusion

In summary, we identified a region related to atopy on chromosome 2p21 that contains *SGK493 *gene. A non-synonymous SNP in *COL18A1*, intronic variants in *MAP3K5 *and a polymorphism in *COL29A1*, previously related to atopic dermatitis, were also associated with atopy, but not consistently replicated. Follow-up analysis in larger sample sets, as well fine mapping will be needed to validate these susceptibility loci.

## Abbreviations

*SGK493*: protein kinase-like protein SgK493; *COL29A1*: collagen, type XXIX, alpha 1; *MAP3K5*: mitogen-activated protein kinase kinase kinase 5; *ASK1*: apoptosis signal-regulating kinase 1; *SLC19A1*: solute carrier family 19 (folate transporter), member 1; *COL18A1*: collagen, type XVIII, alpha 1; GWA: Genome-Wide Analysis; ECRHS: European Community Respiratory Health Survey; IgE: Immunoglobulin E; SNP: single nucleotide polymorphisms; FDR: false discovery rate; HWE: Hardy-Weinberg equilibrium; LD: Linkage disequilibrium; LTD(4): transcription factor activator protein-1 (AP1) in leukotriene D (4); NO: nitric oxygen; kDa: kilodaltons; VEGF: vascular endothelial growth factor; *FCER1A*: Fc IgE receptor, alpha polypeptide.

## Competing interests

The authors declare that they have no competing interests.

## Authors' contributions

FCG participated in the design of the study, prepared the DNA for pooling, performed the statistical analysis and wrote the first draft of the manuscript; MB participated in the design of the second stage of the study, prepared the DNA, performed the genotyping of the individual samples and wrote the first draft of the manuscript; JRG participated in the design of the second stage of the study and assisted with the statistical analysis; MK conceived the study, participated in the design of the study and participated in writing the manuscript; DJ was responsible for design and coordination of the ECRHS study in UK and participated in writing the manuscript; JH was responsible for design and coordination of the ECRHS study in Germany and participated in writing the manuscript; JMA was responsible for design and coordination of the ECRHS study in Spain and participated in writing the manuscript; MW was responsible for DNA biobank, participated in the design of the study and participated in writing the manuscript; XE participated in the design of the study and participated in writing the manuscript; RdC conceived the study, coordinate the study and participated in writing the manuscript; All authors read and approved the final manuscript.

## Pre-publication history

The pre-publication history for this paper can be accessed here:

http://www.biomedcentral.com/1471-2350/10/128/prepub

## Supplementary Material

Additional file 1 Additional file is in acrobat reader file format. Contains 3 tables and 1 Figure: • Additional table S1 - SNPs associated with asthma or atopy with a FDR of 5% and the surrounding region in the pooling-based GWA. • Additional table S2 - Results of the association between the 53 SNPs and asthma or atopy at an individual level. • Additional table S3 - Statistical power calculation for replication using Quanto software v1.2.4 http://hydra.usc.edu/gxe for significance (two-sided) of 0.05 and additive genetic model. Additional figure S1 - Linkage disequilibrium pattern for the HapMap CEPH population of the region flanking SGK493 gene (10 kb at 5' and 3') based on the method of Gabriel et al[18] implemented in Haploview. Click here for file
