# Supplementary material for: A pooling-based genome-wide analysis identifies new potential candidate genes for atopy in the European Community Respiratory Health Survey (ECRHS)
Source: BMC Med Genet. 2009 Dec 6;10:128. doi: 10.1186/1471-2350-10-128 (PMC2797505; doi:10.1186/1471-2350-10-128)
Supplement: Additional file 1 — Additional file is in acrobat reader file format. Contains 3 tables and 1 Figure: • Additional table S1 - SNPs associated with asthma or atopy with a FDR of 5% and the surrounding region in the pooling-based GWA. • Additional table S2 - Results of the association between the 53 SNPs and asthma or atopy at an individual level. • Additional table S3 - Statistical power calculation for replication using Quanto software v1.2.4 http://hydra.usc.edu/gxe for significance (two-sided) of 0.05 and additive genetic model. Additional figure S1 - Linkage disequilibrium pattern for the HapMap CEPH population of the region flanking SGK493 gene (10 kb at 5' and 3') based on the method of Gabriel et al[18] implemented in Haploview. [file 1471-2350-10-128-s1-2.pdf]

Additional tables

Additional table S1 - SNPs associated with asthma or atopy with a FDR of 5% and the surrounding region in the pooling based GWA.

Comparison	Chromosome	Size	Saturation	SNP	Position (build 36)	p value	Region
Atopy vs. controls	1	113 kb	No	rs627897	178153748	0.5775	*TOR1AIP1*
				rs507603	178163693	0.0218	
				rs2777910	178167601	0.0070	
				rs561233	178173523	0.0173	
				rs2501621	178211524	$7.5x10^{-5}$	*CEP350*
				**rs2501618**	**178225432**	**$1.3x10^{-7}$**	***CEP350***
				rs2477106	178231760	5.3**x10**$^{-5}$	*CEP350*
				rs4076449	178263641	0.0119	*CEP350*
				rs12125245	178267165	0.1956	*CEP350*
	2	32 kb	Yes	rs10184755	42112452	0.4742	
				rs11124858	42113828	0.0137	
				rs729559	42126488	1.1 **x10**$^{-5}$	
				**rs4952590**	**42130425**	**$4.6\ x10^{-7}$**	***SGK493 (LOC91461)***
				rs1440099	42144541	0.1391	
	5	126 kb	No	rs7443674	20868696	0.3347	Segmental Dups
				rs10079831	20914020	0.0085	Segmental Dups
				**rs9292961**	**20975886**	**$5.3\ x10^{-7}$**	**Segmental Dups**
				rs3913348	20983113	0.0377	
				rs1979833	20989186	0.0156	
				rs12186507	20994575	0.4109	
	6	39 kb	Yes	rs1011969	137020933	0.0562	*MAP3K5 (ASK1)*
				rs3765259	137031976	0.0119	*MAP3K5 (ASK1)*
				**rs9376221**	**137034072**	**$3.9\ x10^{-7}$**	***MAP3K5 (ASK1)***
				rs9402839	137036903	0.0234	*MAP3K5 (ASK1)*
				rs1009709	137059442	0.1737	*MAP3K5 (ASK1)*
	8	199 kb	Yes	rs12549251	33124129	0.1638	NT_007995.49
				rs6991314	33168920	0.0248	NT_007995.50
				rs7845697	33230933	0.00145	NT_007995.50
				**rs7843085**	**33235086**	**$3.93\ x10^{-7}$**	**NT_007995.50**

				**rs13273924**	**33246541**	**7.5 x10^{-7}**	**NT_007995.50**
				rs6468154	33264445	0.0029	NT_007995.50
				rs10954906	33298381	0.03334	
				rs1996363	33323268	0.61445	NT_007995.51
	21	38 kb	Yes	rs7278425	45750979	0.0580	*COL18A1*
				rs12483377	45755537	0.0418	*COL18A1*
				rs7499	45756756	0.0170	*COL18A1*
				rs3788190	45761386	0.0126	*SLC19A1*
				rs2838956	45769452	0.0002	*SLC19A1*
				**rs2330183**	**45777720**	**1.59 x10^{-7}**	***SLC19A1***
				rs1051266	45782222	0.0029	*SLC19A1*
				rs3788205	45788806	0.1125	
Asthma vs. controls	3	42 kb	Yes	rs9875499	131631200	0.2845	*COL29A1 (COL6A5)*
				**rs7629719**	**131642624**	**1.2 x10^{-7}**	***COL29A1 (COL6A5)***
				rs9883988	131645085	0.0206	*COL29A1 (COL6A5)*
				rs819085	131657024	0.0005	*COL29A1 (COL6A5)*
				rs322117	131673320	0.4556	*COL29A1 (COL6A5)*

SNPs marked in bold are the ones that remain significant after 5 % false discovery rate. *CEP350*: centrosomal protein 350kDa; *SGK493*: protein kinase-like protein SgK493; *COL29A1*: collagen, type XXIX, alpha 1; *MAP3K5*: mitogen-activated protein kinase kinase kinase 5; *ASK1*: apoptosis signal-regulating kinase 1; *SLC19A1*: solute carrier family 19 (folate transporter), member 1; *COL18A1*: collagen, type XVIII, alpha 1.

Additional table S2 - Results of the association between the 53 SNPs and asthma or atopy at an individual level.

					Atopy vs. controls			Atopic asthma subsample vs. controls		
SNP	Gene	MAF	Chromosome	Position	Pooling	Replication	Combined	Pooling	Replication	Combined
**rs11124858***	***SGK493***	**0.369**	**2**	**42113827**	**0.0021**	**0.0317**	**0.0007**[†]	**0.0684**	**0.0666**	**0.0008**[†]
**rs13409978***	***SGK493***	**0.123**	**2**	**42119205**	**0.0007**[†]	**0.0124**	**0.0001**[†]	**0.0054**	**0.0312**	**0.0004**[†]
rs11894613	*SGK493*	0.276	2	42121895			Genotyping under 80%			
rs17029121	*SGK493*	0.269	2	42123362	0.9519	0.8078	0.8212	0.7624	0.5152	0.6484
**rs4952590****	***SGK493***	**0.137**	**2**	**42130424**	**0.0001**[†]	**0.0008**[†]	**1.9×10^{-6}**[†]	**0.0052**	**0.0127**	**3.4×10^{-5}**
rs2424	*SGK493*	0.214	2	42139078	0.4936	0.3135	0.4958	0.5450	0.4572	0.3744
rs12987129	*SGK493*	0.362	2	42141431			Genotyping under 80%			
**rs1440095**	***SGK493***	**0.380**	**2**	**42143169**	**0.0098**	**0.1078**	**0.0105**	**0.0661**	**0.0385**	**0.0018**
rs16845861	*COL29A1*	0.217	3	131581038	0.9836	0.2904	0.3336	0.6657	0.2857	0.2409
rs1453241	*COL29A1*	0.222	3	131586398	0.9829	0.4252	0.4050	0.5530	0.3693	0.2569
rs10212372	*COL29A1*	0.217	3	131586895	0.9328	0.4223	0.3966	0.6160	0.3703	0.2570
**rs10934938**	***COL29A1***	**0.223**	**3**	**131596324**	**0.1158**	**0.0291**	**0.0056**	**0.7549**	**0.0262**	**0.0046**
rs4688761	*COL29A1*	0.281	3	131607678	0.4775	0.5082	0.3616	0.7861	0.4720	0.2250
rs4688801	*COL29A1*	0.074	3	131631449	0.3844	0.8128	0.5813	0.9889	0.7232	0.4292
rs9289373	*COL29A1*	0.082	3	131637553	0.4965	0.7046	0.5712	0.8211	0.9340	0.6802
rs7629719**	*COL29A1*	0.059	3	131642623	0.7569	0.5895	0.6534	0.6123	0.8423	0.9823
rs819085*	*COL29A1*	0.054	3	131657023	0.7569	0.8084	0.7711	0.3056	0.8965	0.7830
rs819084	*COL29A1*	0.083	3	131657717			Genotyping under 80%			
rs819080	*COL29A1*	0.176	3	131660335	0.6432	0.8266	0.9899	0.5522	0.9438	0.9723
rs6791107	*COL29A1*	0.295	3	131667481	0.8673	0.3725	0.3764	0.9123	0.7131	0.5125
rs322114	*COL29A1*	0.125	3	131671837	0.9914	0.1570	0.2215	0.2454	0.1660	0.2174
rs322117	*COL29A1*	0.170	3	131673319	0.7209	0.6677	0.8814	0.9645	0.7288	0.8619
rs2272887	*MAP3K5*	0.147	6	137021826	0.2740	0.9490	0.4390	0.5125	0.9894	0.3207
rs7773600	*MAP3K5*	0.185	6	137026911	0.0833	0.4486	0.1054	0.0653	0.6171	0.1175
rs13195420	*MAP3K5*	0.400	6	137030727	0.7206	0.9375	0.7945	0.5123	0.5894	0.4058
rs3765259*	*MAP3K5*	0.489	6	137031975	0.3265	0.1260	0.0507	0.3635	0.6918	0.4241
**rs9402839***	***MAP3K5***	**0.137**	**6**	**137036902**	**0.0355**	**0.0524**	**0.0099**	**0.6434**	**0.1047**	**0.0322**
rs12198737	*MAP3K5*	0.070	6	137037479	0.6951	0.6210	0.8993	0.1339	0.3399	0.6538

**rs9494554****	***MAP3K5***	**0.095**	**6**	**137038674**	**0.0306**	**0.0035**	**0.0004†**	**0.3989**	**0.0023**	**0.0003†**
rs4363056	*MAP3K5*	0.352	6	137039311	0.2974	0.6139	0.2691	0.3691	0.3535	0.8907
rs11154879	*MAP3K5*	0.239	6	137047059			HWE deviation			
rs7761896	*MAP3K5*	-	6	137048808			Genotyping failed			
rs1009709	*MAP3K5*	0.485	6	137059441	0.1803	0.9757	0.4262	0.9931	0.2221	0.9499
rs12549251	NT_007995.50	0.488	8	33124128	0.0612	0.1442	0.0261	0.3315	0.4325	0.0447
rs16880419	NT_007995.50	0.203	8	33125480	0.8517	0.7315	0.5762	0.4619	0.8333	0.8287
rs17757671	NT_007995.50	0.299	8	33147915			HWE deviation			
rs9656760*	NT_007995.50	0.423	8	33174463	0.0831	0.2092	0.0391	0.1860	0.1212	0.0203
rs10808333	NT_007995.50	0.467	8	33187844	0.0168	0.9024	0.1892	0.2010	0.2386	0.0387
rs11778813	NT_007995.50	0.090	8	33209329	0.2914	0.2554	0.1209	0.3831	0.0730	0.0221
rs11984493	NT_007995.50	0.269	8	33218635	0.0387	0.9835	0.4097	0.9206	0.2015	0.0399
rs7843085**	NT_007995.50	0.297	8	33235085	0.0247	0.8979	0.4243	0.4524	0.1579	0.0312
rs13273924**	NT_007995.50	0.331	8	33246540	0.0117	0.9470	0.2968	0.6208	0.1656	0.0114
rs7000239	NT_007995.50	-	8	33257418			Genotyping failed			
rs12677799	NT_007995.50	0.366	8	33266838	0.3763	0.2675	0.5439	0.7191	0.2668	0.3879
rs4478533	NT_007995.50	0.491	8	33268691	0.2235	0.3038	0.8432	0.2253	0.3548	0.3130
rs3923524	NT_007995.50	0.346	8	33288516	0.3072	0.4134	0.7794	0.7551	0.2088	0.2739
rs7003617	NT_007995.50	0.206	8	33311182	0.3535	0.3722	0.7736	0.2713	0.2684	0.1706
rs11785888	NT_007995.50	0.206	8	33322047	0.6737	0.2474	0.4980	0.9951	0.7946	0.6904
rs1996363	NT_007995.50	0.128	8	33323267	0.3297	0.1643	0.0906	0.2439	0.2666	0.1281
rs7278425	*COL18A1*	-	21	45750978			Genotyping failed			
rs2838951	*COL18A1*	0.412	21	45754147	0.5865	0.2474	0.1554	0.0253	0.5041	0.5845
**rs12483377***	***COL18A1***	**0.103**	**21**	**45755536**	**0.0047**	**0.0607**	**0.0030**	**0.3047**	**0.3392**	**0.0311**
rs2330183**	*SLC19A1*	0.424	21	45777719	0.5685	0.7021	0.4318	0.0025	0.3166	0.2428
rs3788205	*SLC19A1*	0.287	21	45788805	0.68389	0.4089	0.3354	0.2206	0.2985	0.3048

SNPs in bold are the positive ones, listed in Table 1. SNPs marked with asterisk are the ones found associated in the GWA using pools or their perfect tags ($r^2 \geq 0.8$) used as substitutes in the genotyping design Minor allele frequency calculated in the combination of both samples, using control and atopic subjects. * SNPs found associated in the pooling-based GWA. ** SNPs that remained significant after a 5 % FDR in the pooling-based GWA). † SNPs that remain significant after Bonferroni correction ($p \leq 0.001$). *SGK493*: protein kinase-like protein SgK493; *COL29A1*: collagen, type XXIX, alpha 1; *MAP3K5*: mitogen-activated protein kinase kinase kinase 5.

Additional table S3 - Statistical power calculation for replication using Quanto software v1.2.4 (http://hydra.usc.edu/gxe) for significance (two-sided) of 0.05 and additive genetic model.

		Parameters from individual samples from DNA pooling		A priori power		Effective power	
SNP	Gene	MAF	Odds Ratio	Case/control	Power	Case/control	Power
rs11124858*	SGK493	0.367	0.43	429/222	0.99	331/178	0.99
rs13409978*	SGK493	0.122	0.22	429/222	0.99	334/178	0.99
rs4952590**	SGK493	0.141	0.18	429/222	0.99	317/163	0.99
rs1440095	SGK493	0.379	0.50	429/222	0.99	333/172	0.99
rs10934938	COL29A1	0.23	0.57	429/222	0.96	296/150	0.87
rs9402839*	MAP3K5	0.138	2.39	429/222	0.99	314/166	0.99
rs9494554**	MAP3K5	0.095	2.47	429/222	0.99	329/175	0.99
rs12483377*	COL18A1	0.108	4.11	429/222	0.99	282/137	0.99

*SNPs found associated in the pooling-based GWA or their perfect tags ($r2 \geq 0.8$) used as substitutes in the genotyping design.
**SNPs that remained significant after a 5 % FDR in the pooling-based GWA

Additional figure S1 - Linkage disequilibrium pattern for the HapMap CEPH population of the region flanking SGK493 gene (10 kb at 5' and 3') based on the method of Gabriel et al. [1] implemented in Haploview.

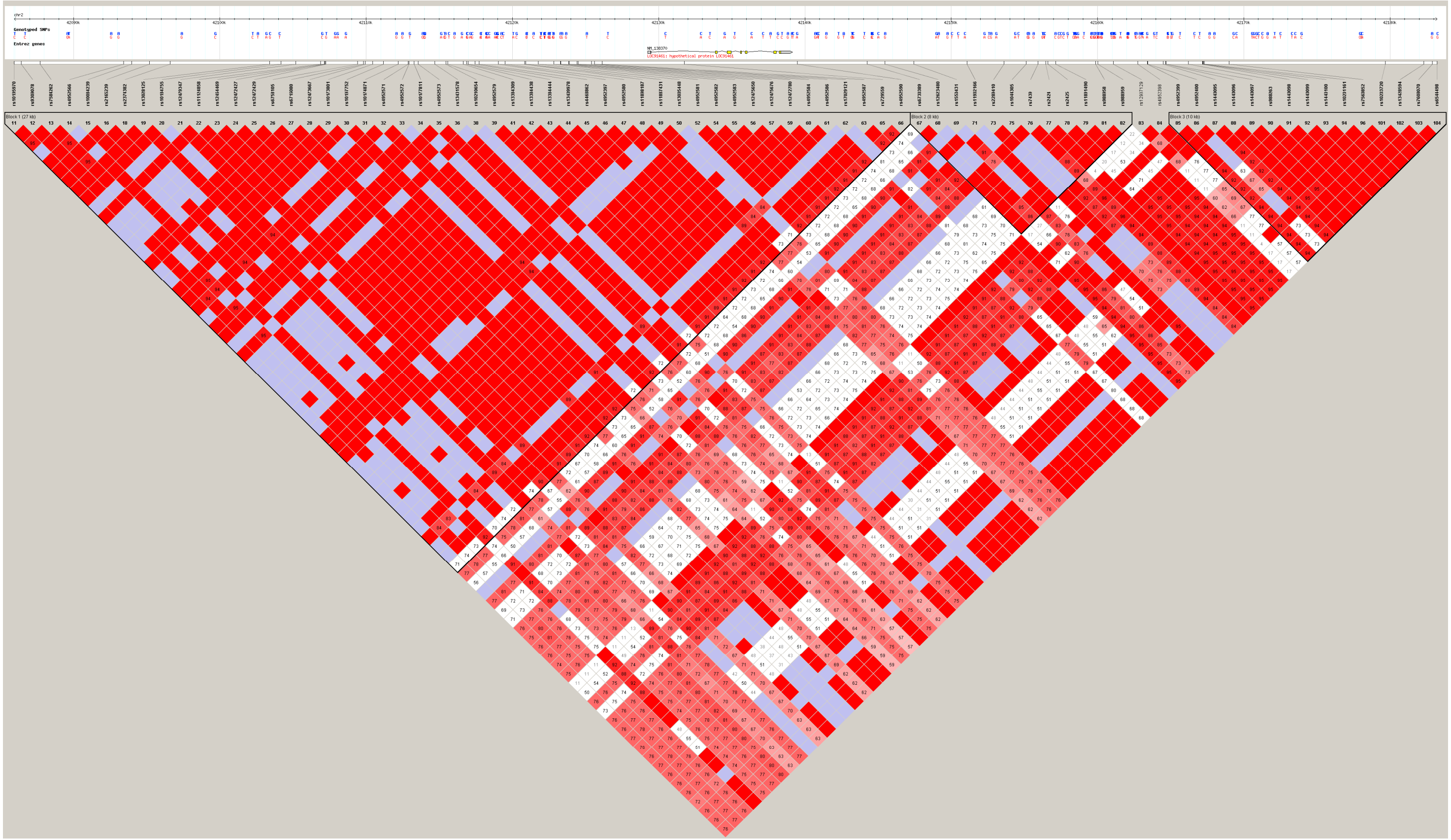

1. Gabriel SB, Schaffner SF, Nguyen H, Moore JM, Roy J, Blumenstiel B *et al.*: **The structure of haplotype blocks in the human genome.** *Science* 2002, 296: 2225-2229.
